# Ultraviolet-irradiated endothelial cells secrete stem cell factor and induce epidermal pigmentation

**DOI:** 10.1038/s41598-018-22608-y

**Published:** 2018-03-09

**Authors:** Misun Kim, Takako Shibata, Soohyun Kwon, Tae Jun Park, Hee Young Kang

**Affiliations:** 1 Department of Dermatology, Ajou University School of Medicine, Suwon, Korea; 2 Shiseido Global Innovation Center, Yokohama, Japan; 3 Department of Biochemistry and Molecular Biology, Ajou University School of Medicine, Suwon, Korea; 4 Chronic Inflammatory Disease Research Center, Ajou University School of Medicine, Suwon, Korea; 5 Department of Biomedical Science, The Graduate School, Ajou University, Suwon, Korea

## Abstract

Ultraviolet (UV)-associated hyperpigmented skins are characterized with increased vasculature underlying pigmentation, suggestive of the possible biological role of endothelial cells in the regulation of skin pigmentation during UV irradiation. In this study, we showed that UV-irradiated endothelial cells significantly increased the pigmentation of melanocytes through epithelial-mesenchymal crosstalk. The stimulatory effect of endothelial cells was further demonstrated using *ex vivo* human skin. RNA sequence analysis and enzyme-linked immunosorbent assay showed that endothelial cells secrete more stem cell factor (SCF) upon UV irradiation than non-irradiated cells. The increased pigmentation elicited by endothelial cells was abrogated following inhibition of SCF/c-KIT signaling. Together these results suggest that endothelial cells are activated upon UV exposure to release melanogenic factors such as SCF, which contributes to the development of skin hyperpigmentation during chronic sun exposure.

## Introduction

The strategic location of the skin as the barrier between the environment and internal milieu determines its critical function in the preservation of body homeostasis, and ultimately organism survival^1^. Epidermal melanocytes synthesize melanin pigments and play an important role in skin pigmentation by absorbing ultraviolet (UV) radiation, thereby serving as a natural sun screen^2^. However, the excessive production of melanin results in hyperpigmentation, which is represented as UV-associated pigmentary disorders, including solar lentigo and melasma.

Skin pigmentation by UV radiation is caused by chemokines and cytokines secreted by cells surrounding melanocytes, such as keratinocytes or fibroblasts. Keratinocytes secrete several paracrine factors, including alpha -melanocyte-stimulating hormone (α-MSH) and endothelin-1, thereby inducing pigmentation and tanning response upon UV irradiation^3^. The role of UV-irradiated fibroblasts in the regulation of skin pigmentation is well studied^4–7^. During UV irradiation, fibroblasts are senescent and produce multiple skin aging-associated secreted proteins (SAASP) as compared to normal fibroblasts. These SAASP include differently expressed secreted factors controlling melanogenesis. In particular, UV-irradiated fibroblasts produce stem cell factor (SCF) or secreted frizzled-related protein-2 (sFRP2), which influence melanogenesis and play a role in pigmentation of observed in solar lentigo or melasma^8–10^.

Aside from keratinocytes and fibroblasts, studies have suggested a possible biological role of endothelial cells in the regulation of skin pigmentation through epithelial-mesenchymal crosstalk. There are clinical examples supporting such a vascular influence on the development of pigmentary disorders^11–13^. The hyperpigmented skins in solar lentigo or melasma was associated with increased vasculature overlying the epidermal pigmentation^12,13^. The increased vasculature was thought to be a consequence of UV irradiation, as the hyperpigmented skin displayed more prominent solar elastosis and increased expression of vascular endothelial growth factor (VEGF), a major angiogenic factor of UV-irradiated skin, as compared with the perilesional normal skin. These findings suggest that the changes in dermal endothelial cells resulting from chronic sun exposure may cause melanocytes to synthesize melanin in the epidermis.

Several recent studies have highlighted the paracrine crosstalk between endothelial cells and melanocytes^14–17^. A recent study reported the regulatory function of melanocytes in vascularization, wherein choroidal melanocytes regulated uveal vascularization through the secretion of fibromodulin^16^. On the contrary, a regulatory function of endothelial cells on melanocytes was discovered. Under physiological conditions, endothelial cells increase pigmentation by releasing endothelin-1 or inhibit pigmentation via transferrin growth factor (TGF)-β or clusterin^14,15,17^. However, how UV irradiation produces changes in endothelial cell secretions in relation to epidermal pigmentation is questionable. We, therefore, investigated the crosstalk between UV-irradiated endothelial cells and melanocytes.

## Results

### Ultraviolet-irradiated endothelial cells induce melanogenesis of melanocytes

**Figure 1:**
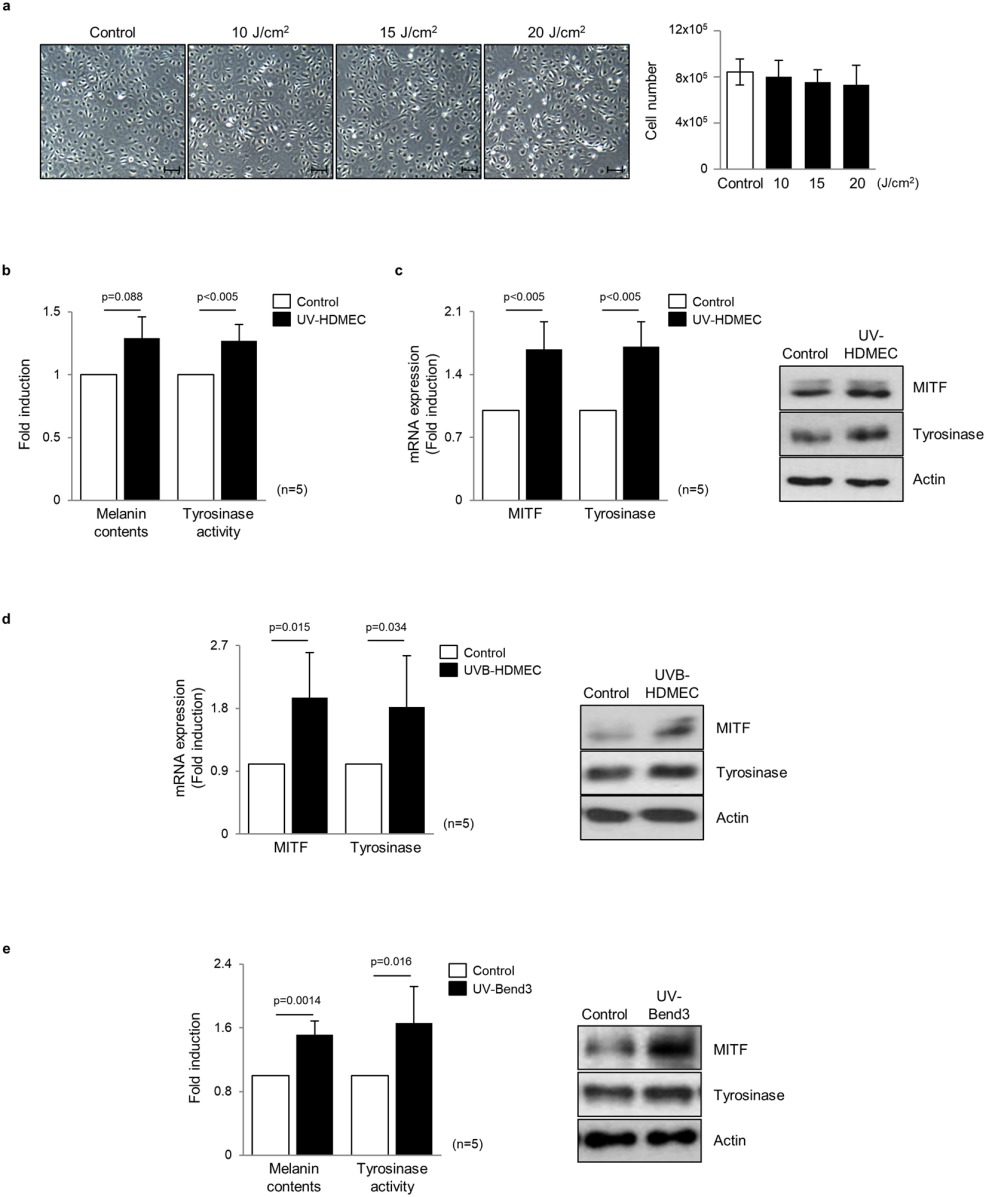
Effects of UV-irradiated endothelial cells on the pigmentation of melanocytes: (**a**) Human endothelial cells (HDMECs) were irradiated with 10–20 J/cm^2^ UVA. After 24 h, the cell morphology was examined and cytotoxicity was analyzed by means of direct counting. (**b**) Normal human melanocytes were treated with a conditioned medium (CM) obtained from 20 J/cm^2^ UV-irradiated HDMECs (UV-HDMECs). Sham-irradiated HDMECs-derived CM was used as control. After 3 days, the melanin content and tyrosinase activity levels were measured. (**c**) The mRNA (left panel) and protein (right panel) expression of MITF and tyrosinase was evaluated using real-time PCR and a western blot analysis, respectively. (**d**) HDMECs were irradiated with 50 mJ/cm^2^ UVB and the CM derived from UVB-HDMECs was used for treatment of melanocytes. The MITF/tyrosinase mRNA (left panel) and protein (right panel) expression levels were then analyzed. (**e**) UV-irradiated Bend3 mouse endothelial cell line-derived medium was applied to B16 melanoma cells and melanogenesis were assessed. All values indicate the mean of independent experiments ± SD. Cropped blots in (**c**–**e**) were displayed and the full-length blots were shown in Supplementary Fig. S9.

Cultured human dermal microvascular endothelial cells (HDMECs) were exposed to ultraviolet A (UVA) at 10–20 J/cm^2 ^^18,19^. The dose of 20 J/cm^2^ was determined as non-cytotoxic energy level according to cell counting (Fig. 1a). To investigate the crosstalk between melanocytes and HDMECs, normal human melanocytes were treated with the conditioned medium (CM) obtained from UV-irradiated HDMECs (UV-HDMECs) and pigmentation assessed. CM derived from sham-irradiated HDMECs was used as control. The results showed that melanin levels and tyrosinase activity were significantly increased in melanocytes treated with CM from UV-HDMECs as compared with the control cells (Fig. 1b, Supplementary Fig. S1). In addition, the mRNA and protein expression levels of melanogenesis-associated proteins, microphthalmia-associated transcription factor (MITF), and tyrosinase were significantly upregulated (Fig. 1c). Melanocyte proliferation was unaffected by either control or UV-irradiated endothelial cells (Supplementary Fig. S2). We also irradiated the cells with UVB and conducted same experiments, as previously reported, to show that UVB irradiation may influence the function of HDMECs^20^. An increase in pigmentation was observed in melanocytes treated with CM from HDMECs irradiated with 50 mJ/cm^2^ UVB, although UVB is known to reach only the endothelial cells located at the superficial dermis level under physiological conditions (Fig. 1d, Supplementary Fig. S3). The stimulatory action of UV-irradiated endothelial cells was further confirmed using UV-irradiated Bend3 mouse endothelial cell line. The treatment of B16 melanoma cells with Bend3-derived CM resulted in a significant increase in the melanin content and melanogenesis (Fig. 1e). To exclude stimulatory effect of the CM on melanogenesis with regard to high tyrosine in DMEM, the cells were cultured in RPMI medium^21^. A stimulatory effect of the Bend3-derived CM on melanogenesis was consistently reproduced (Supplementary Fig. S4).

### Skin pigmentation was induced by UV-irradiated endothelial cells

**Figure 2:**
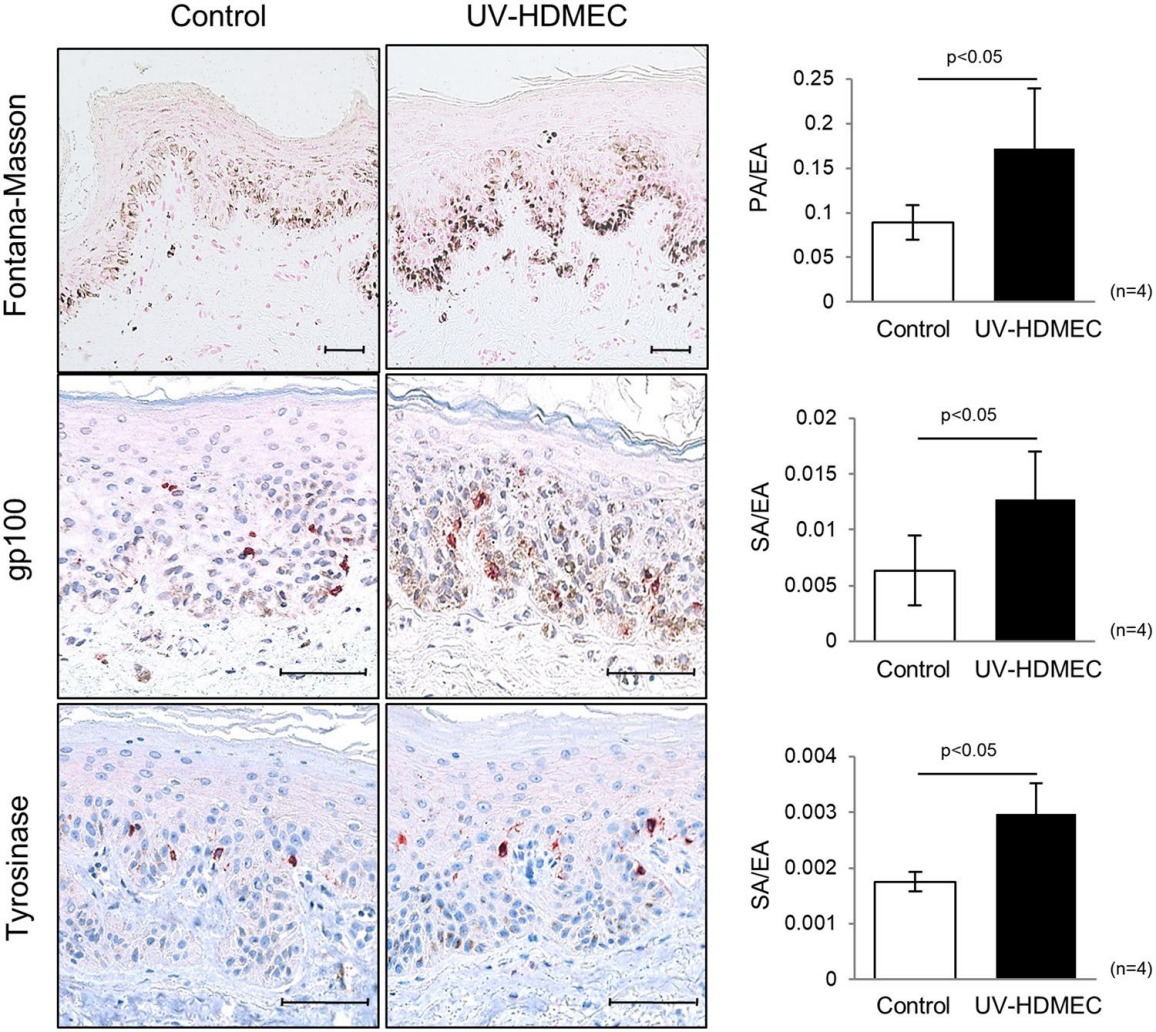
Effects of UV-irradiated endothelial cells on pigmentation of *ex vivo* human skin: *Ex vivo* human skin was maintained with a conditioned medium derived from UV-HDMECs for 3 days. The pigmented area/epidermal area (PA/EA) ratios were measured by image analysis after Fontana-Masson staining. GP100 and tyrosinase expression was detected by immunohistochemical staining and the stained area/epidermal area (SA/EA) ratios were measured. The scale bar indicates 500 μm. All values indicate the mean of independent experiments ± SD.

The stimulatory role of UV-HDMECs on pigmentation was further evaluated using *ex vivo* human skin. The exposure of the skin samples to CM from UV-HDMECs for 3 days resulted in a significant increase in pigmentation as compared with the control (sham-irradiated CM), as observed with Fontana-Masson staining (Fig. 2). Image analysis showed an increase in the pigmented area/epidermal area (PA/EA) ratio for skin samples treated with CM from UV-HDMECs as compared to those treated with control (0.156 ± 0.066 versus 0.084 ± 0.021, n = 4, *p* < 0.05). Immunohistochemical study revealed higher expression of gp100 and tyrosinase in the skin treated with CM from UV-HDMECs as compared with that treated with the control (stained area/epidermal area [SA/EA], 0.014 ± 0.005 versus 0.006 ± 0.003 for gp100 and 0.0018 ± 0.0002 versus 0.0030 ± 0.0006 for tyrosinase, n = 4, *p* < 0.05). Taken together, these data suggest that UV-irradiated endothelial cells induce pigmentation *in vivo*.

### Endothelial cells secrete SCF upon UV irradiation

**Figure 3:**
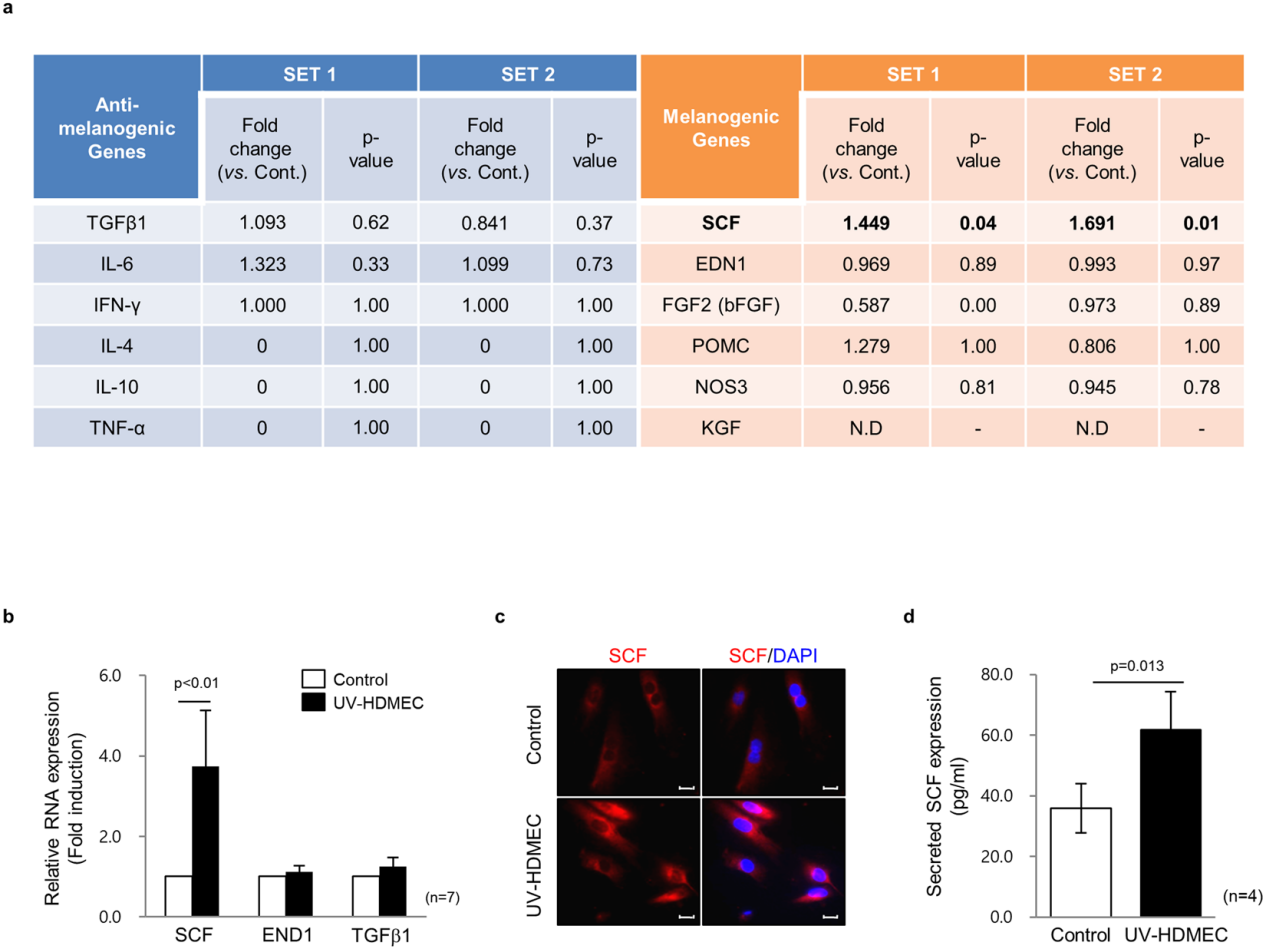
Endothelial cells secrete SCF upon UV irradiation: (**a**) Differential gene expression profiling of HDMECs and UV-HDMECs detected by RNA sequencing analysis. N.D. = none detected. (**b**) Expression levels of the mRNA of SCF, EDN1, and TGFβ1 analyzed by real-time PCR. (**c**) SCF protein expression in the UV-HDMECs detected by immunocytochemical staining. Abbreviation: TGF, transforming growth factor; IL, interleukin; IFN, interferon; TNF, tumor necrosis factor; SCF, stem cell factor; EDN, endothelin; FGF, fibroblast growth factor; POMC, proopiomelanocortin; NOS, nitric oxide synthase; KGF, keratinocyte growth factor. (**d**) Levels of secreted SCF in a cultured medium measured by ELISA. The scale bar indicates 100 μm.

To determine the secreted factors involved in the pigmentation induced by UV-HDMECs, the gene expression profiles of UV-HDMECs were compared to those of sham-irradiated endothelial cells (control). Biostatistical analysis showed that only 131 genes (28 upregulated and 103 downregulated genes, *p* < 0.05) were differentially expressed between UV-HDMECs and control. The majority of the upregulated genes included those involved in cytokine/cytokine receptor interactions and chemokine signaling pathway. We examined the expression levels of various known secreted factors that influence melanogenesis (Fig. 3a). The mRNAs for endothelin-1 (EDN1), proopiomelanocortin (POMC), and basic fibroblast growth factor (bFGF), well-known melanogenic paracrine factors and those for TGF-β, interleukin (IL)-6, and interferon (IFN)-γ (known inhibitory factors) showed no differential expression^1,2,22–24^. Although endothelium is a strong source of nitric oxide (NO) and NO has been shown to induce pigmentation^25,26^, there was no change in the expression of transcripts for NOS. Further, in our co-culture experiments, inhibition of NO with L-NG-nitroarginine methyl ester (L-NMMA, NOS inhibitor) and 2-phenyl-4,4,5,5-tetramethylimidazoline-1-oxyl 3-oxide (PTIO; NO scavenger) did not affect the phosphorylation of MITF (Supplementary Fig. S5). In contrast, the mRNA for SCF showed 1.57-fold higher expression (log2 ratio: 0.646) in UV-HDMECs as compared with the control. Consistent with the gene expression profiling results, real-time polymerase chain reaction (PCR) analysis showed a significant upregulation in SCF level in UV-HDMECs as compared with the control (Fig. 3b). The increase in the expression of SCF protein in these cells was also demonstrated by immunocytochemical staining (Fig. 3c). Furthermore, enzyme-linked immunosorbent assay (ELISA) analysis showed that UV irradiation induced the secretion of soluble SCF in HDMECS as compared with control cells (1.72-fold upregulation, *p = *0.013) (Fig. 3d). The above data were reproduced with UVB experiments and HDMECs were found to secrete more SCF upon UVB irradiation (Supplementary Fig. S6).

### Endothelial cell-derived SCF contributes to skin pigmentation

**Figure 4:**
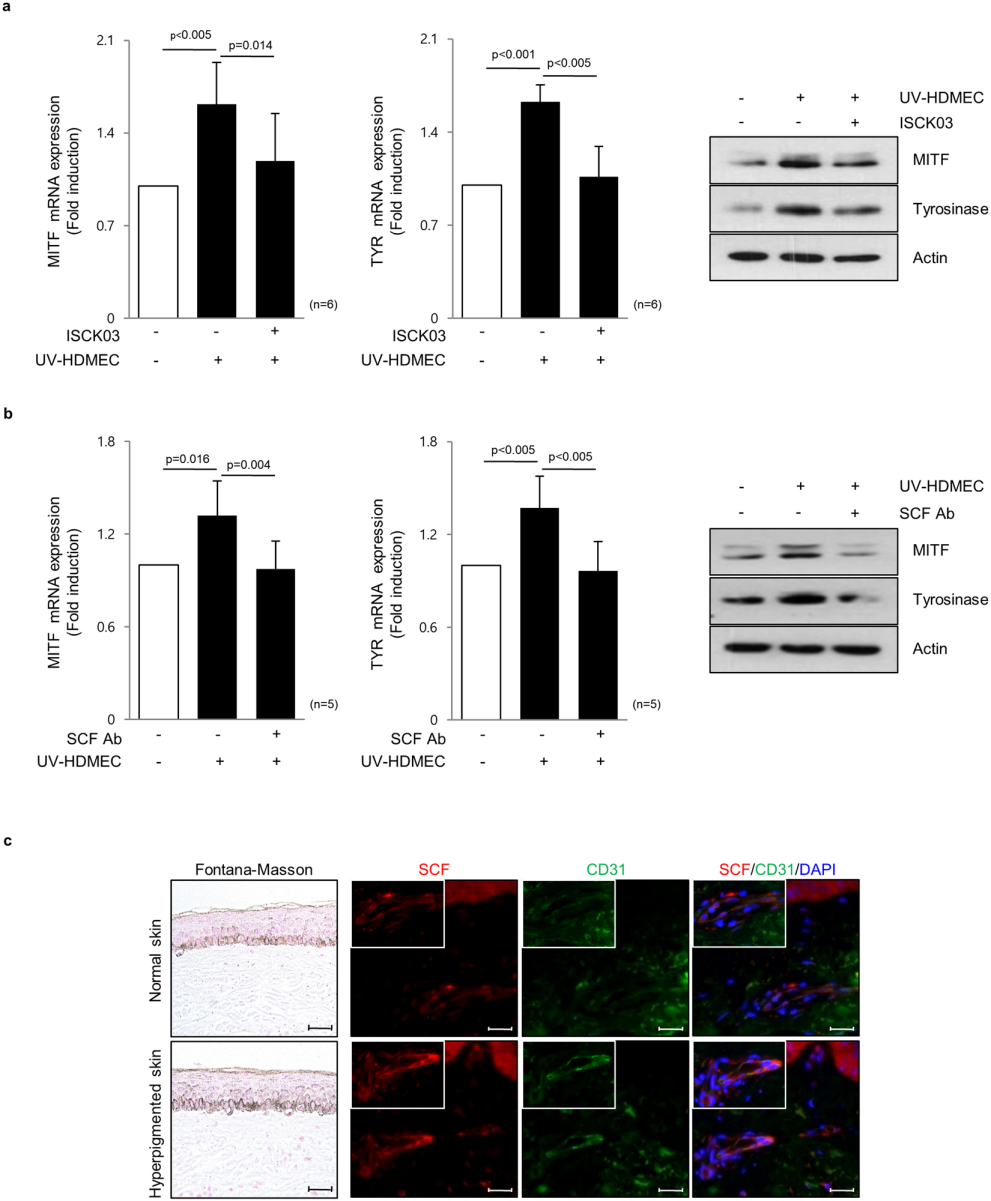
The endothelial cell-derived SCF contributes to skin pigmentation: (**a**) Melanocytes were treated with a KIT-inhibitor, 5 μg/mL ISCK03, in conjunction with a conditioned medium from UV-HDMECs for 3 days. The mRNA (left panel) and protein (right panel) expression levels of MITF and tyrosinase were evaluated using real-time PCR and western blot analysis, respectively. (**b**) A neutralizing anti-SCF antibody (2 μg/mL) was used to block endogenous SCF actions in the conditioned medium from UV-HDMECs. Cropped blots in (**a**,**b**) were displayed and the full-length blots were shown in Supplementary Fig. S10. (**c**) SCF (red) and CD31 (green) double-immunostained endothelial cells were detected in the dermis taken from hyperpigmented skin and normal skin from three melasma patients. All values indicate the mean of independent experiments ± SD. The scale bar indicates 200 μm.

Stem cell factor (SCF) acts as a melanogenic factor through SCF/c-kit signaling in melanocytes^2,27^. To investigate the role of SCF secreted from UV-HDMECs in the modulation of skin pigmentation, melanocytes were treated with a KIT-specific inhibitor (ISCK03) in conjunction with CM from UV-HDMECs. We found that 5 μg/mL ISCK03 was the appropriate concentration required to inhibit the phosphorylation of extracellular-signal-regulated kinase (Erk)1/2, a signaling molecule acting downstream of SCF/c-KIT in melanocytes^28^ (Supplementary Fig. S7), and ISCK03 treatment itself did not affect melanogenesis (Supplementary Fig. S8). The increased pigmentation elicited by UV-HDMECs was abrogated following pretreatment with KIT inhibitor (Fig. 4a). To determine the direct effect of SCF, a neutralizing anti-SCF antibody was used to block endogenous SCF actions in CM from UV-HDMECs. The direct inhibition of SCF significantly diminished the increased mRNA and protein expression levels of MITF and tyrosinase (Fig. 4b). These findings suggested that SCF is the key melanogenic factor secreted from UV-irradiated endothelial cells.

To investigate the role of SCF derived from the endothelial cells in UV-induced pigmentary change *in vivo*, hyperpigmented and normal skin samples were obtained from three melasma patients (Fig. 4c). The analysis of SCF staining revealed a significantly stronger signal in hyperpigmented skin than normal skin. The intensity of CD31 (green)/SCF (red) double-positive cells was much higher in the hyperpigmented skin as compared with the perilesional normal skin. The findings were similar in all three subjects with variable degrees. These results indicate that SCF is upregulated from endothelial cells in UV-induced pigmented skins, suggestive of the role of endothelial cell-derived SCF in the development of hyperpigmentary disorders. The crosstalk between endothelial cells and melanocytes through SCF may contribute to the development of hyperpigmentation of skin.

## Discussion

The present study showed that endothelial cells induce skin pigmentation through the upregulation of SCF during UV irradiation. UV-HDMECs played a stimulatory role in pigmentation and upregulated the expression of melanogenesis regulators, MITF and tyrosinase, in melanocytes. Our results reveal that UV irradiation stimulates endothelial cells to secrete soluble factors, as evident from the increased production of SCF in UV-irradiated endothelial cells as compared with control cells. Moreover, HDMEC-derived SCF played a key role in the paracrine stimulation of melanocytes, thereby increasing the pigmentation of skin. Together these findings suggest the crosstalk between melanocytes and endothelial cells during sun exposure and that this crosstalk plays an important role in the stimulation of melanogenesis and subsequent UV-induced hyperpigmentation.

The association between UV-induced hyperpigmentation and increased vasculature prompted us to use an *in vitro* model of UV-irradiated endothelial cells. The UV irradiation was directly involved in inducing cellular damage and oxidative stress and UV-irradiated endothelial cells were suggested to play an important role in cutaneous aging and various vascular inflammatory disorders^20,29^. In this study, we irradiated endothelial cells with UVA or UVB and clearly showed that UV irradiation directly induced the secretion of soluble SCF, which increased skin pigmentation. The soluble form SCF might be produced by the cleavage of surface bound SCF by several metalloproteases and might be released to the lumen of the vessels as well as secreted into extracellular spaces^30^. Under physiological conditions, endothelial cells are under the influence of UVA rather than UVB. Therefore, further studies should investigate the indirect pathways that function via keratinocytes during UVB irradiation to reveal the relevance of melanogenesis induced by endothelial cells during chronic sun exposure.

Studies have demonstrated the central role of SCF in promoting skin pigmentation by the induction of melanogenesis^2,4,6^. The mRNA and protein expression of dermal SCF and its receptor c-kit was upregulated in the pigmented skin of melasma^6^. The increased epidermal pigmentation after tacrolimus application was thought to be related to the increased expression of SCF in the dermis^31^. In addition, injected SCF was shown to induce pigmentation^32^. All these previous findings highlight the major role of the secreted SCF in increased skin pigmentation and fibroblasts were suggested to be the major cells responsible for increased SCF secretion. Here, we demonstrated additional paracrine crosstalk between endothelial cells and melanocytes to secrete SCF *in vivo*, wherein it played an important role in the modulation of skin pigmentation. We found HDMECs secrete soluble form of SCF and the increase in SCF expression was observed in endothelial cells of pigmented skin *in vivo*. Moreover, we found that the stimulatory effects of UV-HDMECs were prevented by the inhibition of SCF/c-kit signaling, suggestive of the important function of HDMECs-derived SCF in the stimulation of melanocytes and its possible role as a therapeutic target for the development of skin-lightening agents. As the SCF synergizes with myriad growth factors such as interleukins and granulocyte-macrophage (GM)-colony stimulating factor, synergizing effect with other secretory factors requires further investigation^33^.

In summary, this study provides evidence that UV-irradiated endothelial cells produce secreted factors, including SCF, which mediate the stimulatory effect on pigmentation and may play an important role in the pigmentary changes during chronic sun exposure. Further studies are necessary to elucidate the role of other unknown factors during the interaction between endothelial cells and melanocytes.

## Materials and Methods

### Cell culture and treatment

Normal human melanocytes were isolated from 10 human foreskins (average age 10.8 years) during circumcision surgery through a modification of a previously described method^34^. This study was approved by the Institutional Review Board of Ajou University Hospital (AJIRB-GEN-GEN-12-107) and conducted in accordance with the approved human ethics guidelines. All participants were informed of the instructions and procedure of experiments and signed written informed consent forms prior to the experiments. These cells were cultured in F12 medium supplemented with 10% fetal bovine serum (FBS, Gibco-BRL, Bethesda, MD), 24 µg/mL 3-isobutyl-1-methylxanthine, 80 nM 12-O-tetradecanoyl phorbol 13-acetate (TPA), 1.2 ng/mL bFGF, and 0.1 µg/mL cholera toxin (all from Sigma, St. Louis, MO). Cells used for all experiments were between passage 2 and 7. HDMECs (Lonza, Basel, Switzerland) were cultured in an endothelial growth medium (EGM^TM^-2-MV BulletKit^TM^, Lonza). Murine B16 melanoma cells were purchased from American Type Culture Collection (ATCC; Manassas, VA) and Bend3 (murine endothelial cell line) were kindly provided by Prof. Jung YS (College of Pharmacy, Ajou University). These cells were cultured in Dulbecco’s modified Eagle’s medium (DMEM; Gibco-BRL) containing 10% FBS. For inhibition studies, melanocytes were treated with a medium conditioned with UVA-irradiated endothelial cells in conjunction with/without KIT-specific inhibitor (ISCK03) or neutralizing anti-SCF antibody (R&D Systems, Minneapolis, MN). The inhibitor and antibody were pretreated for 2 h and 30 min, respectively.

### Ultraviolet irradiation and preparation of HDMEC-derived CM

HDMECs were washed once with phosphate-buffered saline (PBS) and placed in fresh PBS. The cells were irradiated with 20 J/cm^2^ UVA (wavelength 320–400 nm, maximum peak 350 nm) using LZC-1 photoreactor system (Luzchem Research Inc. Ontario, Canada) or 50 mJ/cm^2^ UVB (wavelength 290–320 nm, maximum peak 311 nm) using TL 20 W/12 RS UV lamps (Philips, Eindhoven, Netherlands). The time of irradiation were 119 min and 61 sec, respectively. Sham-irradiated HDMECs were rinsed and placed into the irradiator box for the appropriate time without UV irradiation. After irradiation, the cells were incubated in EGM for 24 h. The medium was collected and filtered through 0.2 μm filters (Millipore, Billerica, MA) to remove cellular components and debris. The medium was further concentrated by 60-fold with protein concentrators (pore size of 3 kDa, Thermo, Rockford, IL) and 50 µL of the concentrated CM in a total volume of 1.5 mL media was used for the treatment.

### Analysis of the melanin content and tyrosinase activity

The cells were lysed with 0.1 M phosphate buffer (pH 6.8) containing 1% Triton X-100 and protease inhibitor cocktail (Roche, Basel, Switzerland). The protein concentration in the supernatants was measured using Lowry assay system. Pellets were solubilized in 100 μL of 1 N sodium hydroxide (NaOH) for 3 h at 60 °C and the absorbance measured at 490 nm wavelength to analyze the melanin content. The melanin content was calculated from a standard curve using synthetic melanin (Sigma). For the assay of the tyrosinase activity, each sample was incubated with 2 mM L-DOPA (sigma) in 0.1 M phosphate buffer (pH 6.8) for 90 min at 37 °C. After incubation, the tyrosinase activity was measured at 490 nm wavelength.

### *Ex vivo* skin culture and pigmentation assay

Five of human skin samples with average age of 20 were obtained during surgery. This study was approved by the Institutional Review Board of Ajou University Hospital (AJIRB-GEN-GEN-12-107) and conducted in accordance with the approved human ethics guidelines. All participants were informed of the instructions and procedure of experiments and signed written informed consent forms prior to the experiments. The skin samples were cultured as previously described^35^. Briefly, a sterilized stainless steel grid was placed on a culture dish containing DMEM supplemented with 4% FBS. The skin specimens were placed on the stainless steel grid and maintained in an incubator at 37 °C with 5% CO_2_. After 3 days, the specimens were fixed in 10% formalin and embedded in paraffin sections. Melanin pigments were visualized by Fontana-Masson staining. Image analysis was conducted using Image Pro Plus Version 4.5 software (Media Cybernetics Co., Rockville, MD) and the pigmented area relative to the epidermal area (PA/EA) measured. Immunohistochemical staining was performed using antibodies against tyrosinase (1:300 dilution; Invitrogen) and GP100 (1:20 dilution; Monosan, Mountain View, California).

### Immunocytochemical and immunohistochemical analyses

For immunocytochemistry, cells were fixed in 4% paraformaldehyde for 10 min at room temperature and permeabilized with 0.2% Triton X-100. The non-specific binding of the antibody was blocked with 1% bovine serum albumin (BSA) for 1 h, followed by overnight incubation of cells with anti-SCF (Abcam) antibody at 4 °C. Immunohistochemical staining was performed on 4% paraformaldehyde-fixed, paraffin-embedded sections. Antibodies against CD31 (1:75 dilution; Novocastra, Newcastle, UK) and SCF (1:200 dilution; Abcam) were used for protein detection. Informed written consent was obtained from three melasma women patients (average age 43.3 years) before the skin biopsies. The study was approved by the institutional review board of Ajou University Hospital (AJIRB-DEV-DE3-15-491) and conducted in accordance with the approved human ethics guidelines. All participants were informed of the instructions and procedure of experiments and signed written informed consent forms prior to the experiments.

### RNA sequence analysis

Total cellular RNA was extracted from the samples using RNeasy Mini Kits (Qiagen). The quantity and quality of the RNA was measured using a nanophotometer (Implen GmbH, Munich, Germany) and agarose gel electrophoresis, respectively. Total RNA was enriched for mRNA by means of poly(A) selection. Standard Illumina protocols (Illumina, San Diego, CA) were used to generate 2 × 100 bp paired-end read libraries that were sequenced on the Illumina HiSeq. 2500 platform. We used R package DESeq to identify differentially expressed genes^36^. The difference was considered significant if the adjusted *p* value did not exceed 0.05.

### Real-time PCR

Total cellular RNA was extracted using RNeasy Mini kit (Qiagen, Valencia, CA) and the cDNA obtained using SuperScript^TM^ III reverse transcriptase kit (Invitrogen, Waltham, MA). The primer sequences used were as follows: human MITF sense, 5′-AGAACAGCAACGCGCAAAAGAAC-3′ and antisense, 5′-TGATGATCCGATTCACCAAATCTG-3′; human tyrosinase sense, 5′-CACCACTTGGGCCTCAATTTC-3′ and antisense, 5′-AAAGCCAAACTTGCAGTTTCCAC-3′; human SCF sense, 5′-AATCCTCTCGTCAAAACTGAAGG-3′ and antisense, 5′-CCATCTCGCTTATCCAACAATGA-3′; human EDN1 sense, 5′-GACATCATTTGGGTCAACAC-3′ and antisense, 5′-GGCATCTATTTTCACGGTCT3′; human TGFβ1 sense, 5′-GGCGATACCTCAGCAACCG-3′ and antisense, 5′-CTAAGGCGAAAGCCCTCAAT-3′; and human 18S sense, 5′-CGGCTACCACATCCAAGGAA-3′ and antisense, 5′-GCTGGAATTACCGCGGCT-3′.

### Western blot analysis

Cells were harvested and lysed in radioimmunoprecipitation assay (RIPA) buffer (1% NP-40, 150 mM sodium chloride [NaCl], 10 mM Tris-HCl [pH 8.0], and 1 mM ethylenediaminetetraacetic acid [EDTA]) with a complete protease inhibitor (Sigma). The proteins were separated with sodium dodecyl sulfate polyacrylamide gel electrophoresis (SDS-PAGE) and the gel transferred onto a polyvinylidene fluoride membrane (Millipore). Antibody against MITF was purchased from Abcam (Cambridge, UK), while those against tyrosinase, β-actin, and glyceraldehyde 3-phosphate dehydrogenase (GAPDH) were obtained from Santa Cruz Biotechnology (Dallas, TX). Antibodies against p-Erk1/2 and Erk1/2 were provided by Cell Signalling Technology (Danvers, MA).

### Enzyme-linked immunosorbent assay

HDMECs were irradiated with 20 J/cm^2^ UVA or 50 mJ/cm^2^ UVB and cultured in EGM. After 24 h, the conditioned media were harvested. The secreted SCF was measured in the culture media using SCF ELISA kits (R&D Systems), as per the manufacturer’s instructions.

### Statistical analysis

Statistical significance was tested with Mann-Whitney U test (SPSS 22.0; SPSS Inc., Chicago, IL). A value of *p* < 0.05 was considered significant.

## Electronic supplementary material

Supplementary Figure
