# Supplementary material for: Ultraviolet-irradiated endothelial cells secrete stem cell factor and induce epidermal pigmentation
Source: Sci Rep. 2018 Mar 9;8:4235. doi: 10.1038/s41598-018-22608-y (PMC5844989; doi:10.1038/s41598-018-22608-y)
Supplement: Supplementary file 1 — Supplementary Figure [file 41598_2018_22608_MOESM1_ESM.pdf]

Supplementary information

Ultraviolet-irradiated endothelial cells secrete stem cell factor and induce epidermal pigmentation

Misun Kim[1], Takako Shibata[2], Soohyun Kwon[1], Tae Jun Park[3,4,5], Hee Young Kang[1,5]

[1]Department of Dermatology, Ajou University School of Medicine, Suwon, Korea

[2]Shiseido Global Innovation Center, Yokohama, Japan

[3]Department of Biochemistry and Molecular Biology, Ajou University School of Medicine, Suwon, Korea

[4]Chronic Inflammatory Disease Research Center, Ajou University School of Medicine, Suwon, Korea

[5]Department of Biomedical Science, The Graduate School, Ajou University, Suwon, Korea

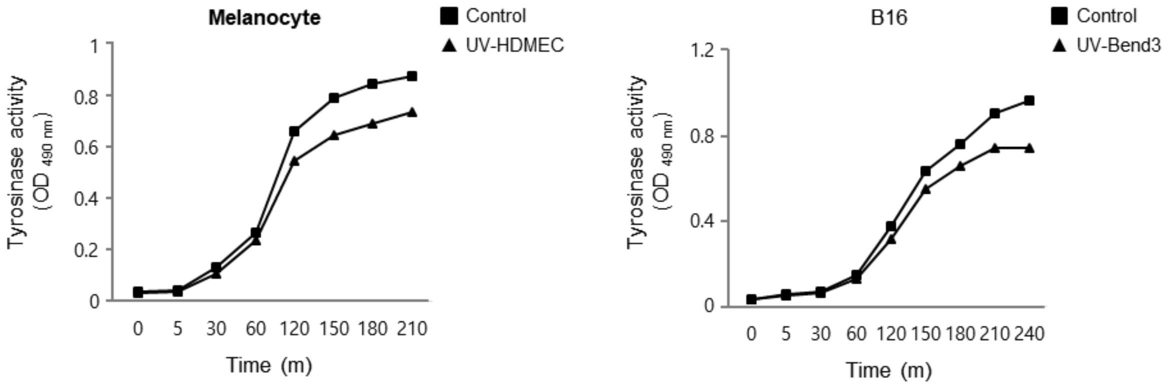

Supplementary Figure 1. The kinetic assay of tyrosinase activity.

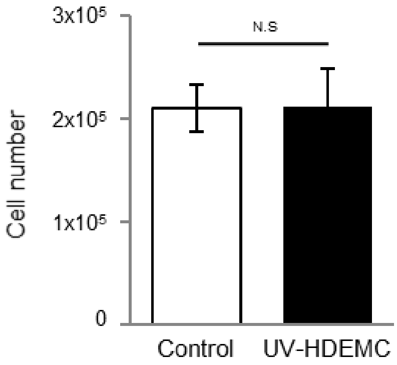

Supplementary Figure 2. UV-irradiated endothelial cells did not affect melanocyte proliferation.

Normal human melanocytes were treated with the conditioned medium (CM) obtained from UV-irradiated HDMECs (UV-HDMECs) and cell proliferation was measured using direct counting. N.S represents no significance.

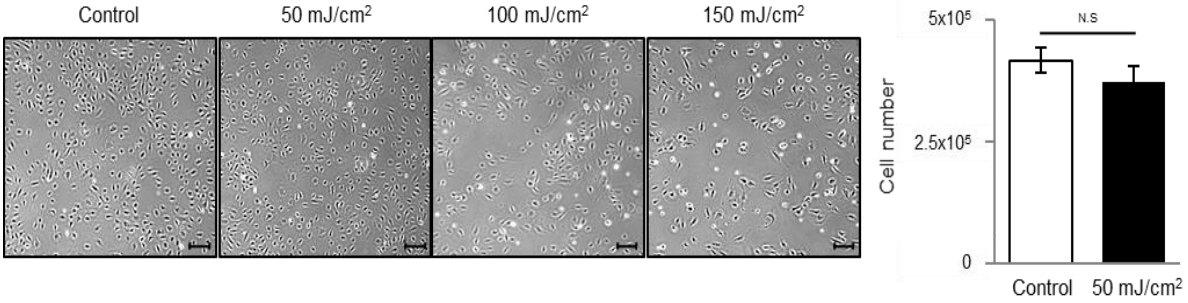

Supplementary Figure 3. The non-cytotoxic level of UVB.

Human endothelial cells (HDMECs) were irradiated with 50-150 J/cm^2 UVB. After 24 h, the cell morphology was examined and cytotoxicity was analyzed by means of direct counting.

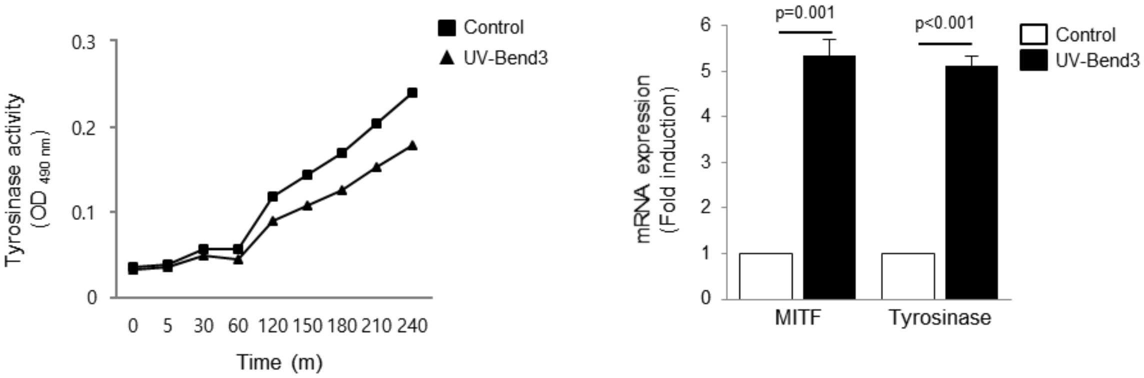

Supplementary Figure 4. Effects of UV-irradiated endothelial cells on the pigmentation of B16 melanoma cells cultured in RPMI medium.

A stimulatory effect of the Bend3-derived conditioned medium on melanogenesis was consistently reproduced in RPMI medium.

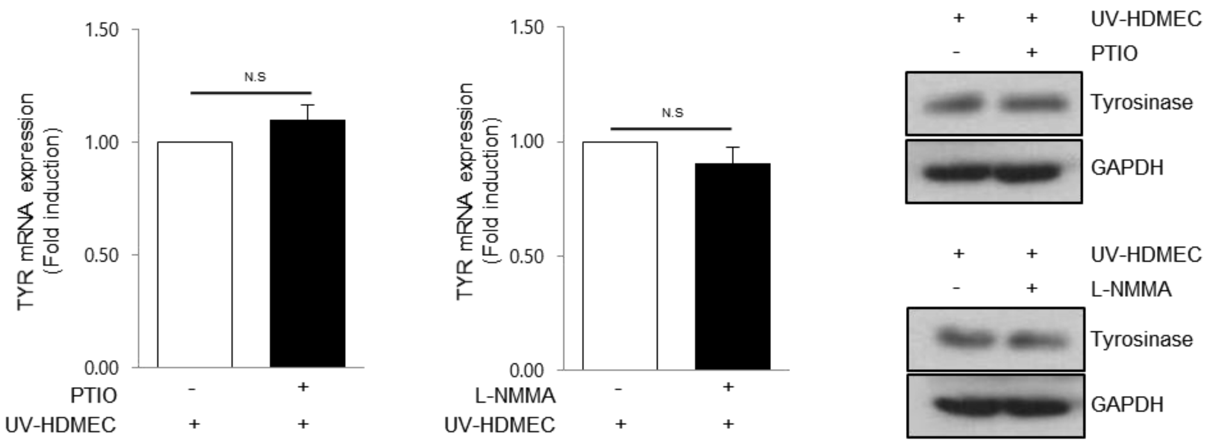

Supplementary Figure 5. Inhibition of NO activation did not affect the pigmentation induced by UV-HDMECs.

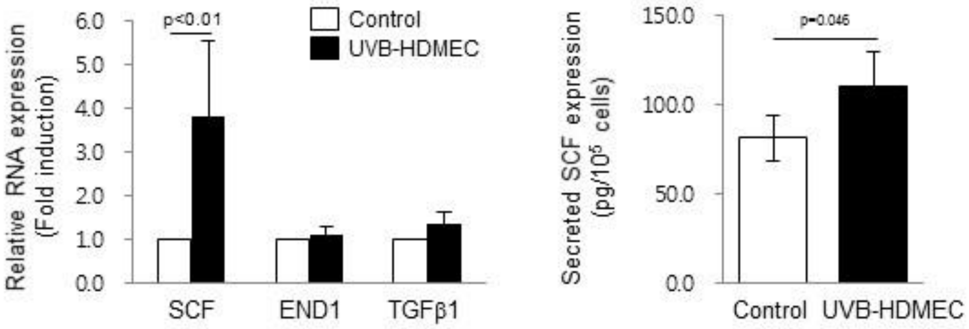

Supplementary Figure 6. Endothelial cells secrete SCF upon UVB irradiation.

Expression levels of the mRNA of SCF, EDN1, and TGFβ1 analyzed by real-time PCR (left panel). Levels of secreted SCF in a cultured medium measured by ELISA (right panel).

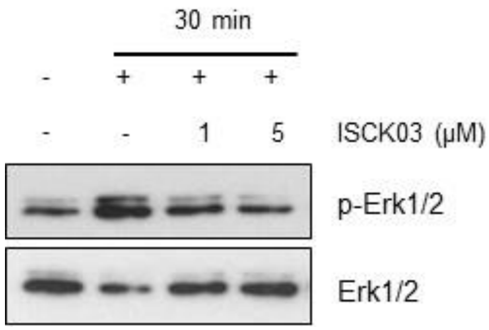

Supplementary Figure 7. The effective dose of ISCK03 in melanocytes.

Melanocytes were treated with a KIT-specific inhibitor (ISCK03) in conjunction with CM from UV-HDMECs. Phosphorylation of extracellular-signal-regulated kinase (Erk)1/2 was analyzed by western blotting. 5 µg/mL ISCK03 was the appropriate concentration required to inhibit Erk1/2 phosphorylation, a signaling molecule acting downstream of SCF/c-KIT in melanocytes.

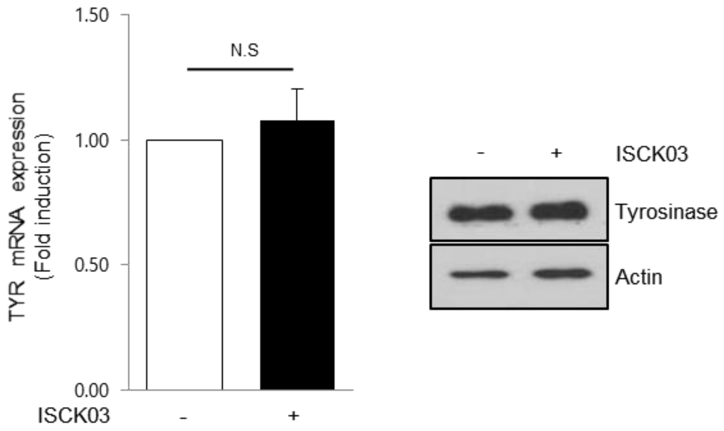

Supplementary Figure 8. Blocking c-kit receptors with ISCK03 treatment did not affect melanogenesis in melanocytes.

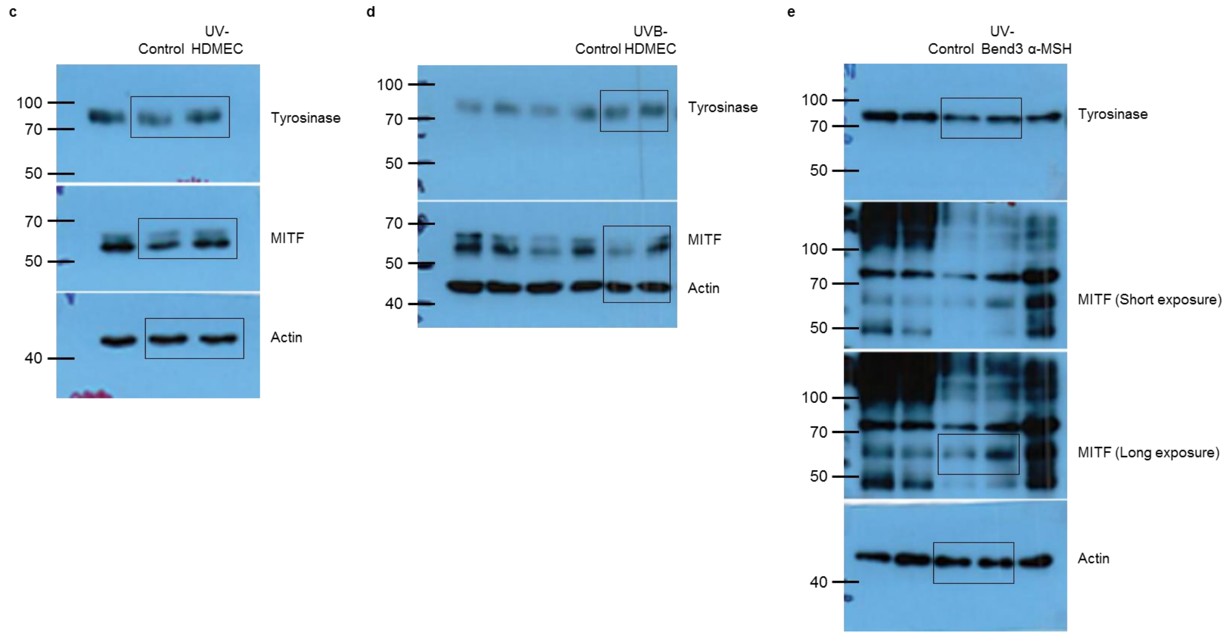

Supplementary Figure 9. Full-length western blots for Figures 1c, 1d, and 1e.

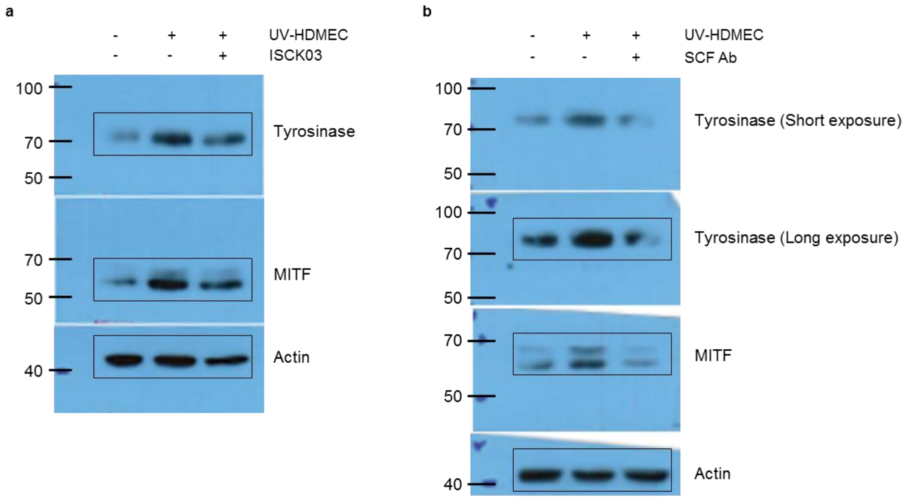

Supplementary Figure 10. Full-length western blots for Figures 5a and 5b.
